# A Fully Automated Chemiluminescent Immunoassay for Component-Resolved Diagnosis of Pollen Allergy: Validation of Six CHORUS CLIA Assays

**DOI:** 10.3390/diagnostics16142158

**Published:** 2026-07-10

**Authors:** Helena Cerutti, Simone Bianciardi, Giulia Barberini, Claudia Soldatini, Alessandra Cartocci, Tommaso Bandini, Giulia Tesi, Alessandra Brogi

**Affiliations:** 1 DIESSE Diagnostica Senese S.p.A Società Benefit, 53035 Monteriggioni, Italy; simonebianciardi@diesse.it (S.B.); giuliabarberini@diesse.it (G.B.); claudiasoldatini@diesse.it (C.S.); tommasobandini@diesse.it (T.B.); giuliatesi@diesse.it (G.T.); alessandrabrogi@diesse.it (A.B.); 2 Department of Molecular and Developmental Medicine, University of Siena, 53100 Siena, Italy; alessandra.cartocci@dbm.unisi.it

**Keywords:** chemiluminescence immunoassay, molecular component, allergen, allergy, validation

## Abstract

**Background/Objectives:** CHORUS CLIA “Allergy Components” is a novel line of fully automated assays designed to detect specific IgE directed against allergenic molecular components. **Methods:** Six singleplex assays—here collectively named as CHORUS CLIA “Allergy Components”—were developed against molecular components of birch (Bet v 1, Bet v 2), timothy grass (Phl p 1, Phl p 5b, Phl p 7), and wall pellitory (Par J 2) and validated on the CHORUS EVO instrument using serum from patients with suspected IgE-mediated sensitization. Limits of detection (LoD) and quantitation (LoQ) were determined according to current guidelines. Comparative analyses of CHORUS CLIA “Allergy Components” with ImmunoCAP ISAC and ImmunoCAP Specific IgE were performed to assess diagnostic accuracy. Spearman’s correlation, Passing–Bablok regression, and Bland–Altman analysis were used to evaluate correlation between methods. **Results:** The LoD was 0.029 kU/L and the analytical interval, 0.10–50.0 kU/L, was validated by LoQ analyses. CHORUS CLIA “Allergy Components” showed high sensitivity (92.0–100.0%) and specificity (96.0–100.0%) versus ImmunoCAP ISAC. Furthermore, diagnostic accuracy was confirmed against ImmunoCAP Specific IgE (Cohen’s kappa: 0.840–1.000). The Spearman coefficient ranged from 0.907 to 0.970 between CHORUS CLIA “Allergy Components” and ImmunoCAP ISAC, and from 0.903 to 0.989 versus ImmunoCAP Specific IgE. The Passing–Bablok analysis showed no differences, and the Bland–Altman analysis revealed outliers only among high-positive samples, without affecting the clinical interpretation. **Conclusions:** The six assays of the CHORUS CLIA “Allergy Components” panel can detect IgE directed against major allergens from *Betula verrucosa* (birch), *Phleum pratense* (timothy grass), and *Parietaria judaica* (wall pellitory). These assays resulted accurate, reproducible, and specific, and showed substantial agreement with both ImmunoCAP ISAC and ImmunoCAP Specific IgE.

## 1. Introduction

Allergens are proteins or glycoproteins capable of inducing IgE production in genetically predisposed individuals. They are commonly categorized by exposure context as indoor (e.g., mites, animal dander, fungi, pests) or outdoor (e.g., pollens, fungal spores), and by exposure pattern as perennial or seasonal. Allergic components are further classified as major or minor according to the proportion of sensitized individuals who recognize them, with major allergens identified by more than 50% of patients sensitized to a given source [1].

Birch, timothy grass, and wall pellitory are major sources of pollen allergens, each with distinct geographic prevalence and clinical impact [2,3,4].

Birch pollen is the dominant tree pollen allergen in Europe, with sensitization rates ranging from 8 to 54% depending on the region [3,5,6]. Bet v 1 represents the principal allergen of birch pollen and accounts for IgE reactivity in a large proportion of birch pollen-allergic individuals [7,8]. Bet v 2, a birch-derived profilin, is considered a minor allergen but exhibits a high degree of structural and sequence homology with profilins from numerous plant species. As a result, it functions as a panallergen and is implicated in extensive cross-reactivity with a broad spectrum of pollens and plant-derived foods [9].

Timothy grass pollen is the leading cause of grass pollen allergy in Europe [10,11]. It contains species-specific allergens (Phl p 1, Phl p 5) and cross-reactive components (Phl p 7, Phl p 12) [12]. Most patients are polysensitized, often with cross-reactivity mediated by profilins and polcalcins [13,14].

Wall pellitory is a major source of allergen exposure in the Mediterranean basin and certain Atlantic regions, accounting for sensitization in up to 50% of allergic individuals in southern Italy [15,16,17]. Its principal allergens, Par j 1 and Par j 2, are well-characterized proteins that share structural features and several IgE-binding epitopes [18]. Genuine sensitization can be distinguished from cross-reactivity (e.g., with mugwort or ragweed) using molecular diagnostics, with Par j 2 serving as a marker for true wall pellitory allergy [4].

While the diagnosis of allergy remains primarily clinical, the evaluation of allergen-specific IgE may help to more accurately define the patient’s sensitization profile and its relationship to the observed clinical manifestations, thus facilitating a more informed therapeutic approach prior to treatment initiation [19]. Molecular allergy diagnostics addresses this need by detecting IgE antibodies directed against specific allergen components [20] using either singleplex or multiplex testing platforms [21,22].

Chemiluminescence immunoassay (CLIA) is increasingly used for quantifying IgE in allergen assays. Its technical features offer several benefits, including the detection of specific IgE at low concentrations [23].

Herein, the term “Allergy Components” refers to a novel panel of fully automated CLIA-based immunoassays for the detection of allergen-specific IgE on the CHORUS platform (Diesse, Monteriggioni, Italy). The assays are performed on the CHORUS EVO instrument, a fully automated immunodiagnostic platform (Diesse, Italy). These assays are commercially available and certified under the In Vitro Diagnostic Regulation (IVDR).

In this study, we evaluated the analytical performance of these assays for the detection of major allergen components from birch, timothy grass, and wall pellitory in human serum samples. Assay performance was assessed through both validation and comparative analyses, using the ImmunoCAP ISAC microarray (Thermo Fisher Scientific Inc., Waltham, MA, USA) and the singleplex ImmunoCAP Specific IgE assays (Thermo Fisher Scientific Inc., USA) as reference methods.

The ImmunoCAP platforms are widely regarded as reference methods in allergy diagnostics. ImmunoCAP ISAC112 is a multiplex microarray system that simultaneously evaluates IgE reactivity to a wide range of molecular allergen components, supporting component-resolved diagnosis. Conversely, the singleplex ImmunoCAP Specific IgE assay quantitatively measures IgE directed against individual allergen extracts or molecular components. Given their extensive clinical validation and routine use, both platforms are commonly adopted as reference methods for the evaluation of new diagnostic assays [21,22].

## 2. Materials and Methods

### 2.1. Serum Samples

Human serum samples were analyzed in this study. The samples consisted of residual sera collected during routine clinical testing at the Allergology Laboratory of the Hospital of Baggiovara (Modena, Italy) and at the Istituto Dermopatico dell’Immacolata (Rome, Italy). The participating institutions provided the samples under a formal collaboration agreement. After collection, sera were stored at −20 °C until analysis. All samples were anonymized prior to use to ensure patient confidentiality.

### 2.2. Test Method

CHORUS CLIA “Allergy Components” identify the series of singleplex chemiluminescent immunoassay designed by DIESSE Diagnostica Senese SpA Società Benefit (Italy) to detect IgE singularly against the following allergens: birch (CHORUS CLIA Bet v 1 82800-T215-BETV1 CHORUS CLIA Bet v 2 82800-T216-BETV2), timothy grass (CHORUS CLIA Phl p 1 82800-G205-PHLP1, CHORUS CLIA Phl p 5b 82800-G215-PHLP5B, CHORUS CLIA Phl p 7 82800-G210-PHLP7), and wall pellitory (CHORUS CLIA Par j 2 82800-W211-PARJ2).

Briefly, an anti-human IgE monoclonal antibody is immobilized on the solid phase. Following incubation with diluted human serum, specific IgE antibodies bind to the capture antibody. Serum samples are diluted in a dedicated buffer containing a Cross-Reactive Carbohydrate Determinant (CCD) blocker to minimize assay-related cross-reactivity due to anti-CCD IgE antibodies potentially present in patients’ sera. After washing to remove unbound material, the solid phase is incubated with the specific biotinylated allergen. Subsequent washing eliminates unreacted proteins, and a streptavidin–peroxidase conjugate is added. After removal of excess conjugate, a luminol-based substrate is introduced, generating a chemiluminescent signal directly proportional to the concentration of allergen-specific IgE in the sample. All reagents required for the assay are contained within disposable test devices. Results are expressed in kilounits per liter (kU/L), calculated using an internal calibration curve integrated into the instrument.

All procedures were performed on the CHORUS EVO instrument (Diesse, Italy), a multiparametric analyzer specifically designed for immunometric assays, including CLIA, which integrates multiple technologies to fully automate the analytical process.

### 2.3. Predicate Device

For comparative evaluation, ImmunoCAP ISAC 112 (Thermo Fisher Scientific Inc., USA) and ImmunoCAP Specific IgE assays (Thermo Fisher Scientific Inc., USA) were conducted according to the manufacturer’s instructions. ImmunoCAP ISAC 112 is a microarray-based assay comprising 112 immobilized molecular allergen components, enabling simultaneous semi-quantitative profiling of IgE reactivity to multiple allergen molecules in a single analysis; analyses are performed on the Phadia 1000 instrument. It is widely used for component-resolved allergy diagnostics and molecular sensitization profiling. ImmunoCAP Specific IgE is a quantitative fluoroenzyme immunoassay routinely employed in clinical allergy diagnostics to measure allergen-specific IgE directed against individual allergenic extracts or purified molecular components.

The comparison study was not planned prospectively (i.e., prior to routine hospital analyses) but was conducted retrospectively on residual samples that were available. Therefore, sample sets differed because they reflected the specific test requests received by the laboratory: most samples had been analyzed using ISAC, whereas only a subset of patients had undergone ImmunoCAP Specific IgE testing. In this comparison, the number of positive samples characterized with the singleplex method was limited, which restricted singleplex analyses to the most prevalent allergens.

### 2.4. Limit of Detection (LoD) and Limit of Quantitation (LoQ)

The limit of detection (LoD) was measured according to the approved guidelineEP17-A2, 2nd edition, of the Clinical and Laboratory Standards Institute (CLSI), with the accuracy goal set at 20% [24,25].

LoD and LoQ were determined on the base device underlying the analytical system using biotinylated IgE calibrated against the international standard for total IgE quantification. These parameters were established at the system level and considered applicable to the entire assay panel, irrespective of the individual allergen. We also analyzed 120 negative samples which were characterized by serum IgE concentrations below the assay’s limit of detection to quantify the analytical background of the test.

Each serum sample was run in triplicate using 2 CHORUS EVO instruments. The homogeneity assumption was used to decide whether the LoD should be calculated using a parametric or a non-parametric approach. Homogeneity was evaluated using the Brown–Forsythe test, as recommended by the guidelines. When homogeneity was confirmed, the parametric approach was applied; otherwise, the non-parametric approach was used. In both cases, the LoD was set as the maximal value obtained for each reagent lot, and the counts per second (cps) value was then transformed into kU/L.

Starting from the LoD concentration, each sample was spiked with IgE at defined interval of concentrations, with approximately 1.5–2-fold dilution between successive values. Reported values correspond to those obtained in the reference test. These concentrations were considered suitable for demonstrating the LloQ, as they effectively illustrated the method’s performance at low levels. Then, starting from the maximum linearity range, each sample was spiked by gradually lowering IgE to defined interval of concentrations, with 14–25% differences between successive values. These concentrations were considered suitable for demonstrating the UloQ, as they effectively illustrated the method’s performance at high levels.

### 2.5. Precision and Repeatability

Precision and repeatability were evaluated using five samples spanning the analytical measuring range. Intra-assay precision was assessed using a single reagent lot on one CHORUS EVO instrument operated by a single user. Each sample was tested in six replicate determinations within the same analytical run.

Inter-assay precision was evaluated using a single reagent lot on one CHORUS EVO instrument over six independent runs in six different days. For inter-lot precision, three different reagent lots were tested on a single CHORUS EVO instrument by the same operator, with each sample analyzed across six runs.

Inter-instrument precision was assessed using a single reagent lot on three CHORUS EVO instruments operated by the same user. Each sample was analyzed over six runs on all three instruments in six different days.

A control sample was included in every analytical run to verify assay performance and confirm run acceptability.

To determine the linearity, 3 samples were tested in duplicate, and the average of the results was plotted on an XY graph. The horizontal axis represented the expected values, and the vertical axis the results obtained. The graph was visually examined to assess the presence of non-linearity and potential outliers.

Analytical specificity was assessed using five serum samples, each tested at defined and progressively increasing concentrations. Bilirubin (4.5–45 mg/dL), triglycerides (250–1500 mg/dL), and hemoglobin (2.5–10 mg/mL) were added to the samples before assays were run and potential interferences were quantified.

### 2.6. Statistical Analysis

Overall accuracy, sensitivity, specificity, positive predictive value, negative predictive value, and Cohen’s kappa coefficient were evaluated in comparison with both ImmunoCAP assays. The comparison with ImmunoCAP ISAC included all allergens detected by CHORUS CLIA “Allergy Components” (Bet v 1, Bet v 2, Phl p 1, Phl p 5b, Phl p 7, and Par j 2), whereas the comparison with ImmunoCAP Specific IgE focused on three allergens: Bet v 1, Phl p 1, and Phl p 5b. Cohen’s kappa values were interpreted as follows: poor (<0.20), fair (0.21–0.40), moderate (0.41–0.60), good (0.61–0.80), and very good (0.81–1.00).

Spearman correlation, Passing–Bablok regression, and Bland–Altman were carried out to determine the correlation between CHORUS CLIA Allergy and both ImmunoCAP assays. A *p*-value < 0.05 was considered statistically significant. All analyses were performed using Analyse-it Software version 5.96 (Analyse-it Software, Ltd., Leeds, UK).

## 3. Results

### 3.1. Validation

Before proceeding with qualitative and quantitative analyses, it is essential to determine the sensitivity and reliability of the assay. Establishing the limits of detection and quantification ensures that the measurements fall within an accurate and reproducible range. Therefore, LoD and LoQ were evaluated to define the detection capabilities for subsequent measurements. The ranges were identical across the entire series of assays, calculated with respect to the quantification of the international total IgE standard. The LoD was 0.029 kU/L, while the lower and upper LoQ values were 0.10 kU/L and 50.0 kU/L, respectively. Accordingly, the validated analytical range for quantitative determination was set between 0.10 and 50.0 kU/L.

Precision, repeatability, and reproducibility were evaluated for all assays and means values obtained for the individual analysis are reported. All assays showed a CV ≤ 15% and were considered acceptable (Table 1).

The analytical evaluation was performed to exclude potential interference from physiological components of human serum. Since the entire series of assays are based on the same analytical device, and the only difference between tests is the specific biotinylated allergen component, the protocol can be evaluated using a single representative parameter, with the results then applicable to the entire series. This is because the only step in which interfering substances may exert an effect is the binding of patient IgE to the solid phase, a step that is identical across all assays. Consequently, the assessment was performed on one representative parameter (Bet v 1). The only out-of-range results were observed with low positive samples spiked with hemoglobin at all the concentrations tested (10 mg/mL, 5 mg/mL, 2.5 mg/mL). All the other obtained results enter within the acceptance range. At the tested concentrations (up to 45 mg/mL and 1500 mg/dL, respectively), bilirubin and triglycerides did not influence the assay results. However, the presence of high hemoglobin levels (>5 mg/mL) may interfere with the accuracy of negative and low-value samples (Supplementary Data).

### 3.2. Quantitative Measures of Diagnostic Accuracy

In the comparison between CHORUS CLIA “Allergy Components” and ImmunoCAP ISAC, between 90 and 170 sera were analyzed. The assay showed high sensitivity (90.2–100.0) and specificity (96.00–100.00) across all allergens tested (Bet v 1, Bet v 2, Phl p 1, Phl p 5b, Phl p 7, Par j 2). Cohen’s k values were similarly high, ranging from 0.92 for Bet v 1 to 0.98 for Bet v 2, Phl p 5b, and Phl p 7, indicating strong agreement between the two assays. High sensitivity and specificity were also observed in comparison with ImmunoCAP Specific IgE for Bet v 1, Phl p 1, and Phl p 5b. Nevertheless, concordance remained high, with Cohen’s k values ranging from 0.840 to 1.000 (Table 2).

**Figure 1:**
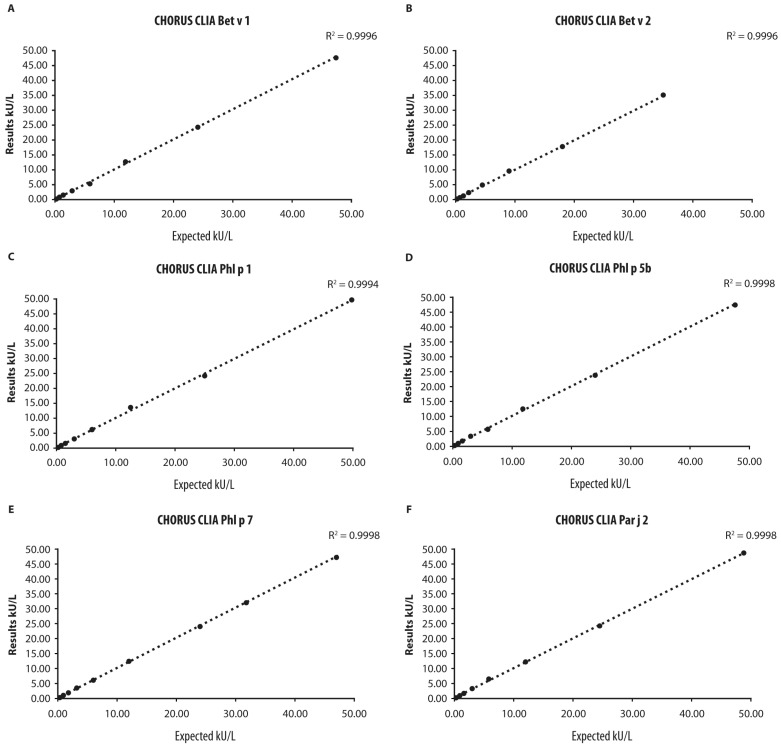
(**A**–**F**) Linearity of CHORUS CLIA “Allergy Components” assays. The graphs represent the linearity for each allergen comparing CHORUS CLIA “Allergy Components” kit line. The R^2^ values are reported for each graph.

No significant deviations from linearity were observed for any of the assays (Figure 1).

### 3.3. Quantitative Analysis

In the quantitative analysis, the strength of the monotonic relationship between the two assays was assessed using Spearman correlation. The results demonstrated excellent agreement: the Spearman coefficient ranged from 0.907 to 0.970 for the comparison between CHORUS CLIA “Allergy Components” and ImmunoCAP ISAC, and from 0.903 to 0.989 for the comparison with ImmunoCAP Specific IgE (Table 3).

**Figure 2:**
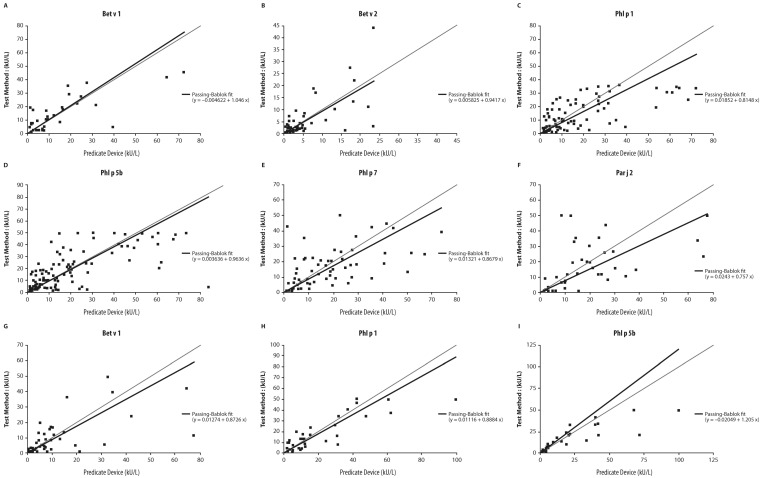
(**A**–**I**) Passing–Bablok analysis. The graphs represent the Passing–Bablok analysis for each allergen comparing CHORUS CLIA “Allergy Components” kit line with ImmunoCAP ISAC (in the upper panel) or ImmunoCAP Specific IgE (in the lower panel). The equations are reported for each graph.

The Passing–Bablok analysis did not show any significant differences between the two assays, as indicated by the 95% confidence interval for the intercepts and slopes (Table 3 and Figure 2). Similarly, no systematic errors or significant differences were observed in the comparison between CHORUS CLIA and ImmunoCAP Specific IgE.

**Figure 3:**
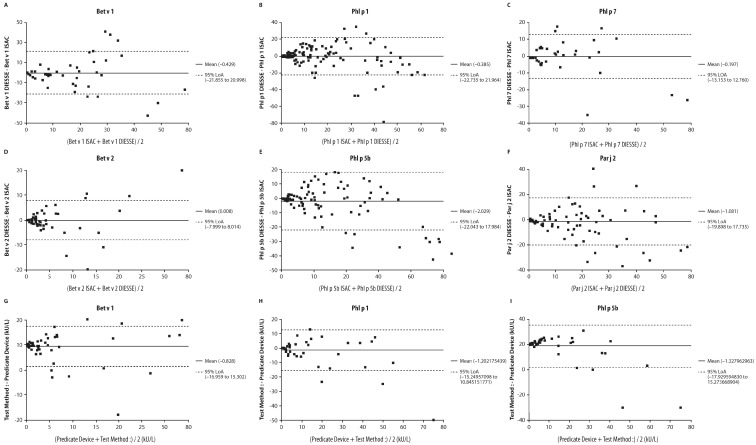
(**A**–**I**) Bland–Altman analysis. The graphs represent the Bland–Altman analysis for each allergen comparing CHORUS CLIA “Allergy Components” kit line with ImmunoCAP ISAC (in the upper panel) or ImmunoCAP Specific IgE (in the lower panel). Outliers were revealed among high-positive samples.

Bland–Altman analysis showed that deviations between CHORUS CLIA “Allergy Components” and both ImmunoCAP assays were confined to samples with very high positive values, and these differences would be expected to have minimal impact clinical decision-making or result interpretation. These quantitative differences could be attributed to the different analysis methods (Figure 3).

## 4. Discussion

This study reports that the six assays, here collectively referred to as CHORUS CLIA “Allergy Components”, provide sensitive, specific, and accurate quantification of IgE directed against birch, timothy grass, and wall pellitory allergens in human sera.

The assay’s detection range spanned from 0.10 kU/L to 50.0 kU/, and it may be suitable for potential clinical applications. The assays demonstrated robust analytical performance, as common serum interferents, including bilirubin and triglycerides, did not affect the results.

The diagnostic accuracy of CHORUS CLIA “Allergy Components” in terms of specificity, sensitivity, and concordance was evaluated against both a multiplex platform (ImmunoCAP ISAC) and a singleplex assay (ImmunoCAP Specific IgE). Quantitative analyses, including Spearman correlation and Passing–Bablok regression, indicated a substantial concordance between CHORUS CLIA “Allergy Components” and both ImmunoCAP methodologies. Overall, CHORUS CLIA “Allergy Components” proved reliable, with no evidence of systematic error or bias relative to the reference assays.

As ImmunoCAP ISAC (expressed in ISU-E, semiquantitative) and CHORUS CLIA (expressed in kU/L, quantitative) use different measurement scales and are not directly comparable in absolute terms, the aim of the statistical comparison (Passing–Bablok regression and Bland–Altman analysis) was not to demonstrate metrological equivalence between units, but rather to assess the presence of systematic bias or proportional deviations (fixed or proportional bias) between the two methods in identifying major allergen sensitizations.

In this frame, the interpretation of the analyses focuses on diagnostic agreement and the detection of potential systematic errors, rather than on the absolute comparability of raw numerical values. The observed differences thus reflect the intrinsic technological characteristics of the two analytical platforms and their respective output scales. We also specify that no mathematical conversion between ISU-E and kU/L units was performed.

As a direct singleplex-to-singleplex comparison would have been preferable to strengthen the evaluation against a fully quantitative method, a comparison with ImmunoCAP Specific IgE (singleplex) was included. This additional analysis was intended to support the suitability of the CHORUS method when compared with a reference operating on a directly comparable quantitative scale (kU/L versus kU/L).

However, this approach was only partially feasible due to the limited number of suitable samples available. Indeed, while the collaborating clinical centers had an adequate number of specimens previously analyzed with ImmunoCAP ISAC, the number of samples positive for ImmunoCAP Specific IgE was more restricted. For certain allergens (Bet v 2, Par j 2, and Phl p 7), it was not possible to obtain enough positive samples, largely due to differences in clinical testing practices.

Taken together, the available data still allowed an evaluation of concordance with both reference systems, considering the known correlation between ImmunoCAP ISAC and ImmunoCAP Specific IgE.

A distinctive feature of our assay is its targeted design, which focuses on a specific panel of allergens (Bet v 1, Bet v 2, Phl p 1, Phl p 5b, Phl p 7, Par j 2). This panel includes the main clinically relevant molecular allergens for respiratory allergy in Europe, encompassing both major sensitizers such as birch (Bet v 1), timothy grass (Phl p 1, Phl p 5b), and wall pellitory (Par j 2), as well as pan-allergens relevant to cross-reactivity, such as profilins (Bet v 2) and polcalcins (Phl p 7). The selection is consistent with the recommendations of international consensus documents on molecular allergy diagnostics (WAO/ARIA/GA^2^LEN PAMD@, 2020) [26]. By focusing on clinically relevant allergens and including minor but strongly cross-reactive components such as Bet v 2, this approach ensures comprehensive assessment while reducing analytical ambiguity [19,27].

CHORUS CLIA “Allergy Components” assays employ a fully automated workflow that allows the analytical procedure to be completed within a defined timeframe. This configuration may facilitate their use in routine laboratory practice, including in settings where more complex instrumentation is not accessible. Performing the entire procedure on the CHORUS EVO platform contributes to workflow standardization and may help limit operator-related variability.

The main limitation of the study was the lack of clinical patient data that would allow for framing the results in a clinical context. However, the assays of the CHORUS CLIA “Allergy Components” proved to be reliable, accurate, and comparable to ImmunoCAP ISAC and ImmunoCAP Specific IgE, which are currently used in clinical practice. Future studies linked to clinical outcomes could provide valuable additional insights.

## 5. Conclusions

The six assays of the CHORUS CLIA “Allergy Components” panel were able to detect IgE directed against major allergens from birch, timothy grass, and wall pellitory, which are recognized sources of pollen sensitization in several European regions. These assays provided accurate, reproducible, and specific results, and showed substantial agreement with both ImmunoCAP ISAC and ImmunoCAP Specific IgE. Future expansion of the panel could allow the evaluation of a broader range of allergenic sources.

## Figures and Tables

**Table 1:** Precision assay. A coefficient of variation (CV) ≤15% was considered acceptable.

Precision CV%				
KIT	Within-Run	Between-Run	Between-Batches	Between-Instruments
CHORUS CLIA Bet v 1	4.2	8.6	6.1	9.6
CHORUS CLIA Bet v 2	6.7	12.8	7.9	14.1
CHORUS CLIA Phl p 1	6.3	7.4	7.4	8.1
CHORUS CLIA Phl p 5b	3.4	9.3	7.6	11.4
CHORUS CLIA Phl p 7	5.2	8.6	5.6	12.6
CHORUS CLIA Par j 2	2.2	4.7	4.5	4.2

**Table 2:** Quantitative measures of diagnostic accuracy. The table summarizes the results of the comparison between the CHORUS CLIA “Allergy Components” kit line and ImmunoCAP ISAC and ImmunoCAP Specific IgE. For each allergen, sensitivity, specificity, agreement, positive predictive value, negative predictive value, and Cohen’s k were reported. Cohen’s K indicated high agreement between the assays.

**Comparison Between CHORUS CLIA “Allergy Components” Kit Line and ImmunoCAP ISAC**									
**KIT**	**Samples**			**Sensitivity**	**Specificity**	**Agreement**	**Positive Predictive Value**	**Negative Predictive Value**	**Cohen’s k**
	**N**	**Pos**	**Neg**						
CHORUS CLIA Bet v 1	100	41	59	90.2	100.0	96.0	100.0	93.7	0.920
CHORUS CLIA Bet v 2	105	52	53	98.1	100.0	99.0	100.0	98.1	0.980
CHORUS CLIA Phl p 1	170	117	53	96.6	100.0	97.6	100.0	93.0	0.950
CHORUS CLIA Phl p 5b	140	91	49	97.8	100.0	98.6	100.0	96.1	0.980
CHORUS CLIA Phl p 7	90	33	57	97.0	100.0	98.9	100.0	98.3	0.980
CHORUS CLIA Par j 2	120	70	50	100.0	96.0	98.3	97.2	100.0	0.960
**Comparison Between CHORUS CLIA “Allergy Components” Kit Line and ImmunoCAP Specific IgE**									
CHORUS CLIA Bet v 1	100	53	47	90.6	93.6	92.0	94.1	89.8	0.840
CHORUS CLIA Phl p 1	95	42	53	100.0	100.0	100.0	100.0	100.0	1.000
CHORUS CLIA Phl p 5b	90	35	55	100.0	98.2	98.9	97.2	100.0	0.980

**Table 3:** Quantitative analysis. The table summarizes the results of the comparison between CHORUS CLIA “Allergy Components” kit line and both ImmunoCAP ISAC and ImmunoCAP Specific IgE. The Spearman coefficient was reported for each allergen, and a strong correlation was highlighted between the assays; all Spearman values showed *p* < 0.001. Concerning the Passing–Bablock analysis, both the slope and the intercept with the corresponding 95% confidence intervals (95% CI) were described.

**Comparison Between CHORUS CLIA “Allergy Components” Kits and ImmunoCAP ISAC**					
**KIT**	**Spearman** **Coefficient**	**Passing–Bablok**			
		**Slope**	**95% CI**	**Intercept**	**95% CI**
CHORUS CLIA Bet v 1	0.965	0.757	(0.524 to 1.070)	0.024	(−0.007 to 0.048)
CHORUS CLIA Bet v 2	0.941	0.942	(0.641 to 1.371)	0.006	(−0.037 to 0.036)
CHORUS CLIA Phl p 1	0.907	0.964	(0.852 to 1.142)	0.004	(−0.014 to 0.015)
CHORUS CLIA Phl p 5b	0.919	0.815	(0.628 to 1.009)	0.019	(−8.734 to 0.037)
CHORUS CLIA Phl p 7	0.970	1.046	(0.646 to 1.322)	−0.005	(−0.032 to 0.035)
CHORUS CLIA Par j 2	0.934	0.868	(0.626 to 1.046)	0.013	(−0.005 to 0.037)
**Comparison Between CHORUS CLIA “Allergy Components” Kits and ImmunoCAP Specific IgE**					
**KIT**	**Spearman Coefficient**	**Passing Bablok**			
		**Slope**	**95% CI**	**Intercept**	**95% CI**
CHORUS CLIA Bet v 1	0.903	0.873	(0.649 to 1.126)	0.013	(−0.013 to 0.035)
CHORUS CLIA Phl p 1	0.987	0.889	(0.633 to 1.184)	0.011	(−0.018 to 0.037)
CHORUS CLIA Phl p 5b	0.989	1.205	(0.985 to 1.523)	−0.02	(−0.052 to 0.001)

## Data Availability

The original contributions presented in this study are included in the article/Supplementary Material. Further inquiries can be directed to the corresponding author.

## Supplementary Materials

The following supporting information can be downloaded at https://www.mdpi.com/article/10.3390/diagnostics16142158/s1, Supplementary Data: Quantitative interference data.

## Abbreviations

The following abbreviations are used in this manuscript:

CI	Confidence Interval
CLIA	Chemoluminescent Immunoassay
Ig	Immunoglobulin
LoD	Limit of Detection
LoQ	Limit of Quantitation
