# Supplementary material for: A Fully Automated Chemiluminescent Immunoassay for Component-Resolved Diagnosis of Pollen Allergy: Validation of Six CHORUS CLIA Assays
Source: Diagnostics (Basel). 2026 Jul 10;16(14):2158. doi: 10.3390/diagnostics16142158 (PMC13408629; doi:10.3390/diagnostics16142158)
Supplement: Supplementary file 1 [file diagnostics-16-02158-s001.zip › diagnostics-4258885-supplementary.pdf]

Supplementary Data: Quantitative interference data

Hemoglobin									
**Sample**	**Level 1 (10 mg/mL)**			**Level 2 (5 mg/mL)**			**Level 3 (2.5 mg/mL)**		
	**Control KU/L**	**Spiked KU/L**	**%diff**	**Control KU/L**	**Spiked KU/L**	**%diff**	**Control KU/L**	**Spiked KU/L**	**%diff**
**N1**	<0.10	<0.10	**0**	<0.10	<0.10	**0**	<0.10	<0.10	**0**
**P1**	0.26	0.68	**162**	0.26	0.49	**88**	0.26	0.34	**31**
**P2**	0.86	1.22	**42**	0.86	0.94	**9**	0.86	0.96	**12**
**P3**	34.95	36.24	**4**	34.95	35.64	**2**	34.95	35.57	**2**

Bilirubin									
**Sample**	**Level 1 (45 mg/mL)**			**Level 2 (18 mg/mL)**			**Level 3 (4.5 mg/mL)**		
	**Control KU/L**	**Spiked KU/L**	**%diff**	**Control KU/L**	**Spiked KU/L**	**%diff**	**Control KU/L**	**Spiked KU/L**	**%diff**
**N1**	<0.10	<0.10	**0**	<0.10	<0.10	**0**	<0.10	<0.10	**0**
**P1**	0.26	0.28	**8**	0.26	0.29	**12**	0.26	0.30	**15**
**P2**	0.86	0.85	**1**	0.86	0.91	**6**	0.86	0.93	**8**
**P3**	34.95	34.66	**1**	34.95	35.01	**0**	34.95	36.03	**3**

Triglycerides									
**Sample**	**Level 1 (1500 mg/dL)**			**Level 2 (750 mg/dL)**			**Level 3 (250 mg/dL)**		
	**Control KU/L**	**Spiked KU/L**	**%diff**	**Control KU/L**	**Spiked KU/L**	**%diff**	**Control KU/L**	**Spiked KU/L**	**%diff**
**N1**	<0.10	<0.10	**0**	<0.10	<0.10	**0**	<0.10	<0.10	**0**
**P1**	0.26	0.26	**0**	0.26	0.28	**8**	0.26	0.23	**12**
**P2**	0.86	0.87	**1**	0.86	0.87	**1**	0.86	0.82	**5**
**P3**	34.95	36.74	**5**	34.95	34.58	**1**	34.95	35.80	**2**
